# Supplementary material for: Transcriptomic Analysis of Respiratory Tissue and Cell Line Models to Examine Glycosylation Machinery during SARS-CoV-2 Infection
Source: Viruses. 2021 Jan 8;13(1):82. doi: 10.3390/v13010082 (PMC7827443; doi:10.3390/v13010082)
Supplement: Supplementary file 1 [file viruses-13-00082-s001.zip › Supplementary Tables_Figures/Figure legends for Supplementary Figures.docx]

**Figure legends for Supplementary Figures:**

Supplementary Figure 1: Reacfoam – DEGs from the SARS-CoV-2 infected human nasopharyngeal swab: Reacfoam shows a high-level comprehensive pathway overview of the differentially expressed glycogenes identified from the SARS-CoV-2 infected human nasopharyngeal swab based on Voronoi tessellation. Significantly enriched pathways are shown in dark blue and the least represented in light green color.

Supplementary Figure 2: Blood group system biosynthesis pathway overlayed with the DEGs identified from the SARS-CoV-2 nasopharyngeal swab samples: A representative pathway figure selected from the Reactome database overlayed with the differentially expressed glycogenes data from the nasopharyngeal swab of SARS-CoV-2 infected patients. The figure represent Blood group system biosynthesis pathway and highlight the association of differentially regulated fucosyltransferases, sialyltransferases and galactosyltransferases with the synthesis of blood group antigens.
