# Supplementary figures and images for: Transcriptomic Analysis of Respiratory Tissue and Cell Line Models to Examine Glycosylation Machinery during SARS-CoV-2 Infection
Source: Viruses. 2021 Jan 8;13(1):82. doi: 10.3390/v13010082 (PMC7827443; doi:10.3390/v13010082)

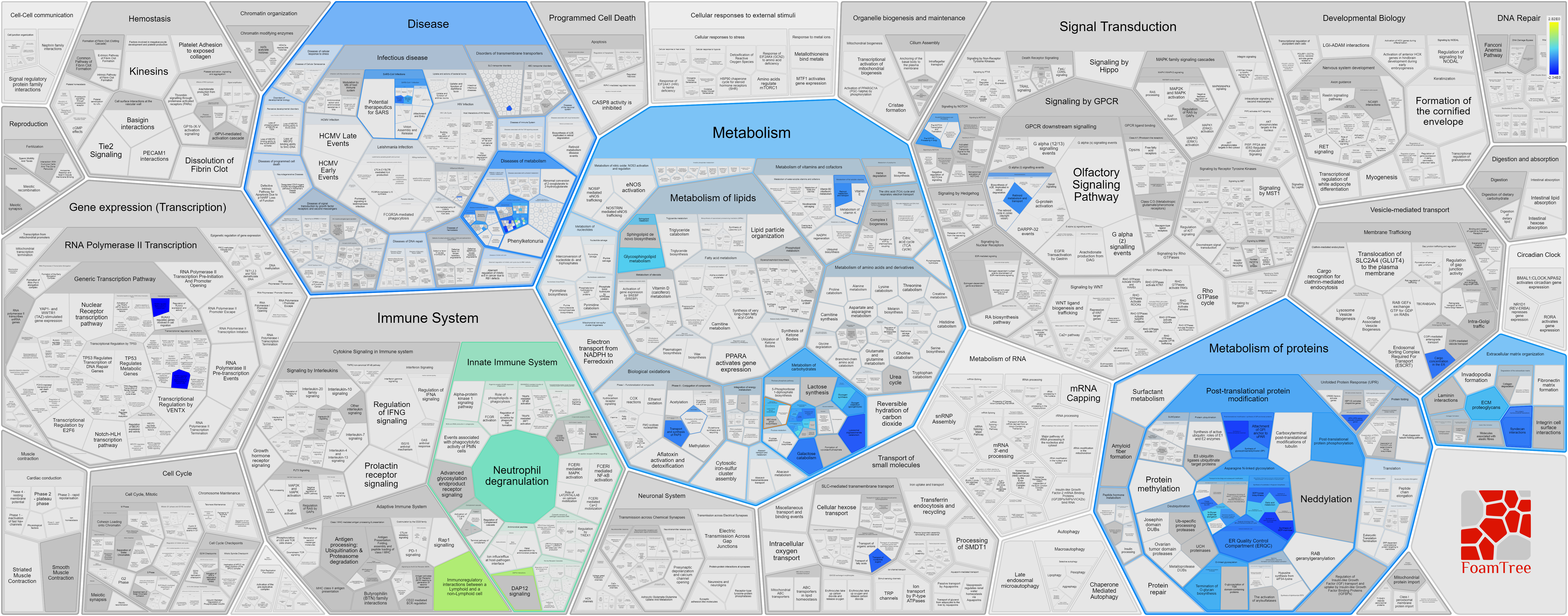

Supplement: Supplementary file 1 [file viruses-13-00082-s001.zip › Supplementary Tables_Figures/Supplementary Figure 1.jpg]

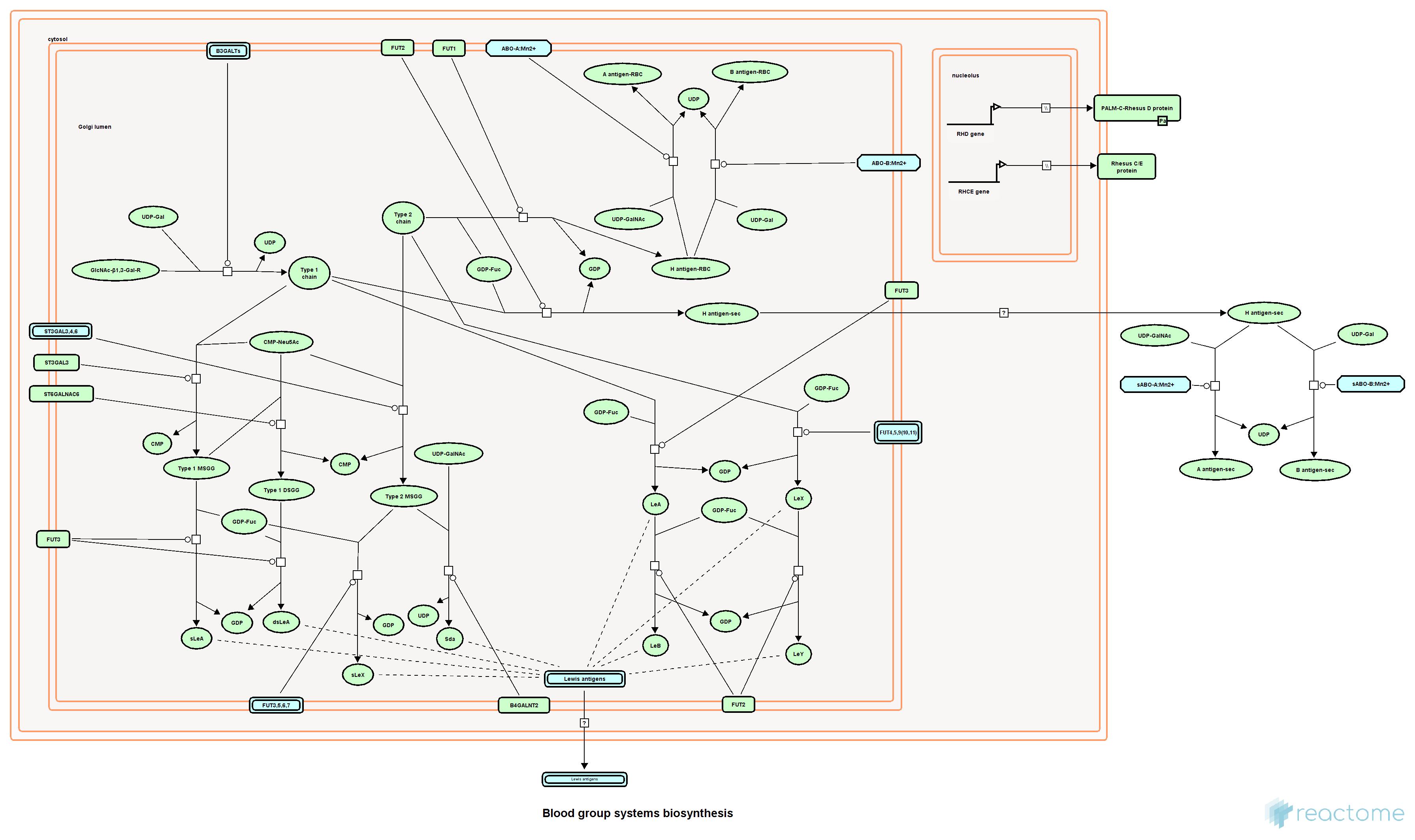

Supplement: Supplementary file 1 [file viruses-13-00082-s001.zip › Supplementary Tables_Figures/Supplementary Figure 2.jpeg]
